# Anxiety and Depression during the Second Wave of the COVID-19 Pandemic: The Role of Coping Strategies

**DOI:** 10.3390/ijerph20042974

**Published:** 2023-02-08

**Authors:** Alessandro Miola, Stefano Caiolo, Giancarlo Pontoni, Erica Pozzan, Chiara Moriglia, Filippo Simionato, Sergio Garofalo, Giulia Perini, Fabio Sambataro

**Affiliations:** 1 Department of Neuroscience (DNS), Padua Neuroscience Center, University of Padova, 35127 Padua, Italy; 2 Medicine Faculty, University of Padova, 35127 Padua, Italy; 3 Casa di Cura Parco dei Tigli, 35037 Teolo, Italy; 4 Psychiatry Section, Military Department of Forensic Medicine, 35137 Padua, Italy; 5 Psychiatry Section, Psychophysiological Selection Office, Italian Army National Recruitment and Selection Center, 06034 Foligno, Italy; 6 Psyops Development Center, 28th (APICE) Regiment “Pavia”, 61121 Pesaro, Italy

**Keywords:** anxiety, depression, affective symptoms, COVID-19 pandemic, coping strategies

## Abstract

Background: Evidence suggests increased anxious-depressive symptoms in the general population during the COVID-19 pandemic, also in its second wave. High symptom variability across individuals suggests that risk and protective factors, including coping strategies, can play a mediating role. Methods: General Anxiety Disorder-7, Patient Health Questionnaire-9, and Brief-COPE questionnaires were administered to people attending a COVID-19 point-of-care. Univariate and multivariate methods were used to test the association of symptoms with risk and protective factors. Results: A total of 3509 participants (27.5% with moderate-severe anxiety; 12% with depressive symptoms) were recruited. Sociodemographic and lifestyle factors, including age, sex, sleep, physical activity, psychiatric treatments, parenthood, employment, and religiosity were associated with affective symptoms. Avoidant (self-distraction, venting, behavioral disengagement) and approach (emotional support, self-blame but not positive reframing and acceptance) coping strategies predicted greater anxiety. Avoidant strategies, including venting, denial, behavioral disengagement, substance use, and self-blame, and the humor strategy were associated with more severe depressive symptoms, while the planning predicted the opposite. Conclusions: Coping strategies, in addition to socio-demographic and life-habit factors, could have contributed to modulating anxious and depressive symptoms during the second-wave of the COVID-19 pandemic, thus advocating for interventions aimed at promoting positive coping strategies to reduce the psychosocial toll of the pandemic.

## 1. Introduction

The psychological and psychiatric consequences of COVID-19 have been extensively explored by a growing number of investigations during the pandemic period. Our group and others have found a 49% prevalence of mild-to-severe COVID-19-related peritraumatic distress in subjects who tested positive for SARS-CoV-2 [1,2,3]. Furthermore, peritraumatic distress associated with COVID-19 has been shown to predict not only posttraumatic stress disorder (PTSD) but also depressive and anxious disorders [4], and, in general, an increased risk of psychopathology [1,2]. Although the entire population underwent the same pandemic crisis, its devastating effects on mental health varied greatly between individuals, suggesting that individual resilience, including coping strategies, could have contributed to mitigating the severe consequences of peritraumatic stress [3]. Coping strategies are “cognitive and behavioral efforts to master, reduce, or tolerate the internal and/or external demands that are created by the stressful transaction” [5]. In other words, “coping” refers to a broad set of strategies used to minimize the effect of stress or adverse events [6]. These behavioral, cognitive, and emotional strategies can be distinguished into approach and avoidant for the orientation toward the stressor [7]: approach coping, when the individual moves actively toward the threat (e.g., seeking information, problem-solving, etc.); avoidant coping, when the individual is passive to the threat or moves away from it physically or emotionally (e.g., denial, distraction, distancing, etc.,). The first strategy is generally effective in reducing psychopathology [8], while the second has a maladaptive value [9].

A previous cross-sectional study conducted during the first phase of the COVID-19 pandemic found that adaptive coping strategies did not influence the levels of depressive and anxiety symptoms, with only self-distraction, within the maladaptive coping strategies being a risk factor for anxiety and depressive symptoms [10]. As the authors reported, these findings were likely influenced by the severe impact of the pandemic and the rapid containment measures that were implemented [10]. Furthermore, some important clues supporting the hypothesis that symptoms of the anxiety-depressive spectrum may be associated with avoidant coping strategies came from studies carried out on specific populations for their relationship with the pandemic, including medical students and healthcare professionals [6,11,12,13]. Unfortunately, in this sample, the excessive increase in workload may have confounded proper pandemic-related psychological distress in the development of psychopathology. Similarly, other investigations focused on samples with specific demographic characteristics, including young adults or women, and this may have affected the generalizability of their results [13,14,15,16,17,18].

Although the second wave of the pandemic has had a negative impact on psychological well-being with a still relevant risk perception, people seemed to adapt to the exposure to the virus by increasing their self-efficacy and internal locus of control [19]. However, confirmatory studies in this regard are still lacking. Finally, several studies investigating psychological well-being during the pandemic were also conducted with an exclusively web-based methodology [10,18,20]. There have been some concerns about the generalizability of findings obtained from online surveys primarily related to selection bias or poor methodological clarity [21,22,23].

Therefore, the current study aims to investigate the association between affective symptoms in the general population and coping strategies that individuals are assigned to prevent and mitigate the psychopathological and social burden associated with the second wave of the COVID-19 pandemic. To minimize the risk of biases of web surveys, we recruited participants directly at the vaccination point.

## 2. Materials and Methods

### 2.1. Participants

A total of 3509 participants were enrolled at a COVID-19 vaccination point in Padua, Veneto, northeast of Italy. This population consisted of individuals who were scheduled to receive an anti-COVID-19 vaccine. Exclusion criteria were age below 18 years and any mental condition that prevented free consent. All 3509 participants completed the questionnaire.

### 2.2. Study Design

Participants were enrolled between June 2021 and January 2022, during the “second wave” of the pandemic, marked by the beginning of the vaccination campaign for the general population and the enforcement of social control measures such as the “green pass” (equivalent to EU Digital COVID Certificate), an entry permit for public places, venues, event or facilities for vaccinated people and patients recently recovered from SARS-CoV-2 infection.

The current study was approved by the local Ethics Committee and performed following the guidelines of the Helsinki Declaration of 1975. All participants provided their informed written consent to participate in the study after a comprehensive explanation of the procedures. All participants received an informative brochure and were asked to complete an anonymous questionnaire. Participants were allowed to complete the survey at any time.

### 2.3. Measures

The informative brochure provided to all participants consisted of a QR code and a link to access the questionnaire, which consisted of a questionnaire on sociodemographics, life habits, and the rating scales for anxiety, depression, and coping styles.

Sociodemographic, life habits, and clinical items. Sociodemographic factors including age, sex, marital status, family composition, educational level, and current occupation were collected. In addition, religious attitudes, alcohol consumption, changes in sleep, and pandemic-related occupational habits were recorded. Moreover, current psychotropic drug and psychotherapy treatments were also investigated. Finally, the main clinical features were gathered for confirmed cases of COVID-19 and symptomatic subjects, including restrictive public health and social measures (RPHSM) in the previous ten days in participants and family members.

Anxiety and depressive symptoms. Anxiety symptoms were assessed using the General Anxiety Disorder-7 (GAD-7) scale [24], a 7-item self-report questionnaire that evaluates the frequency of anxiety, worry, fear, and irritability occurring during the previous two weeks on a 4 -point Likert scale from 0 to 3. A total score of 10–14 and 15–21 can suggest moderate and severe anxiety, respectively [24,25].

Depressive symptoms were measured with the Patient Health Questionnaire-9 (PHQ-9) [26], a 9-item self-report questionnaire that investigates symptoms of a major depressive episode according to DSM-IV criteria in the previous two weeks using a 4-point Likert scale ranging from 0 to 3. Depression is suspected when all the following conditions are present: total score ≥ 10, 5 or more items with a score ≥ 2, of which at least one item must be depressed mood or anhedonia [27].

Coping. Brief COPE, a self-administered 28-item scale, was used to assess coping styles along 14 two-item subscales [7,28]. Coping style is classified into three strategies that include the subscales: avoidant coping, approach coping, and neither/or, but other models have also been proposed [5,7,29,30]. Avoidant coping consists of the following subscales: denial, substance use, venting, behavioral disengagement, self-distraction, and self-blame. Approach coping encompasses the following subscales: active coping, positive reframing, planning, acceptance, seeking emotional support, and seeking informational support. Humor and religion are considered neither avoidant nor approaching strategies [5,7,29,30]. An alternative model of grouping the Brief-COPE subscales further subdivided approach strategies into cognitive vs. emotional, thus differentiating in problem-focused coping that included planning, active coping, positive reframing, and use of informational support; emotion-focused coping that entailed acceptance, self-blaming, venting, seeking emotional support, humor and religion; avoidant coping that encompassed self-distraction, use of substances, behavioral disengagement and denial [31]. Since participants were asked to indicate a score from 1 to 4 for each item, a coping strategy was considered present when scoring at least 6 out of 8 on a specific sub-scale. Given the lack of specific cut-off values for the Brief-COPE subscales, we used these criteria that match the expected 75° centile.

### 2.4. Statistical Analysis

For descriptive statistics, data were summarized by frequency, proportions, percentages, contingency tables for binary variables (i.e., sex, hospitalization); mean, median, range, interquartile range (IQR), variance, standard deviation (SD), confidence interval (95% CI) for continuous variables (i.e., age, PHQ-9 score); median and mode respectively for categorical ordinal and nominal data. For logistic regression analyses, the response variable, GAD-7, and PHQ-9 scores were dichotomized using the cut-off scores for moderate-to-severe anxious symptoms and the PHQ criteria for depression, respectively. Specifically, for GAD-7, we adopted the cutoff score of 10 for moderate to severe anxiety. For PHQ-9, we used the concurrent fulfillment of all three criteria for depressive symptoms (total score ≥ 10; 5 or more items with a score ≥ 2; and either “depressed mood” or “anhedonia” items score ≥ 2). First, all non-binary covariates included in the regression analysis were dichotomized; each of the 14 coping strategies assessed by the Brief-COPE questionnaire was dichotomized as present or absent based on a score of 6 or higher. As expected, this cut-off value represented the best approximation to the 75° centile (mean value 5.7). Subsequently, through univariate logistic regression, the association was explored between the presence of anxious or depressive symptoms and sociodemographics, life habits, clinical characteristics, and coping strategies. Then, two multivariate logistic regression models were built with all factors that reached a *p* < 0.20 at the univariate analysis [32]. Significant predictor and protective factors for anxious and depressive symptoms were selected using the stepwise-backward elimination method. In the multivariate analysis, a *p* < 0.01 was set to identify a significant association between the outcome variable and the covariates. Odds ratios (OR) were used as a measure of effect size. For the power analysis, see the supplementary materials. Statistical analyses were carried out using the STATA Ver. 14.2 software.

## 3. Results

### 3.1. Socio-Demographic Features

Overall, 3509 individuals participated in the current study. The main socio-demographic characteristics are summarized in Table 1. The mean age was 42.2 years (SD = 16.0), and 53.8% were women.

Regarding occupational status, most of the sample had a job (66%), 19% were students, 6.5% were unoccupied, and 8.5% were retired. More than half of the sample reported no changes in their working status (54%), 41% switched to total or partial remote work, and 5.3% lost their job due to the COVID-19 pandemic.

### 3.2. Life Habits, COVID-19 Impact, and Current Psychiatric Treatment

The most relevant life habits and their changes are summarized in Table 1. Most of the participants reported unmodified working time (55%), night sleep (62%), physical activity (42%), and alcohol consumption (74%).

The indirect health-related impact of the diagnosis of COVID-19 was high, with 29% of the participants with (at least) a relative hospitalization and 15% with (at least one) dead person for the consequences of COVID-19.

Current psychopharmacological treatment was regular in 5.7% and sporadic in 7.2%, while psychotherapeutic treatment was reported only in 10.7% of the participants.

### 3.3. Anxiety and Depression

The GAD-7 average score was 6.8 (SD = 5.3), with moderate in 16.5% and severe anxiety in 11% of cases. The average PHQ-9 score was 6.1 (SD = 5.5), and 12% of the participants met the depression criteria.

### 3.4. Coping Strategies

**Figure 1:**
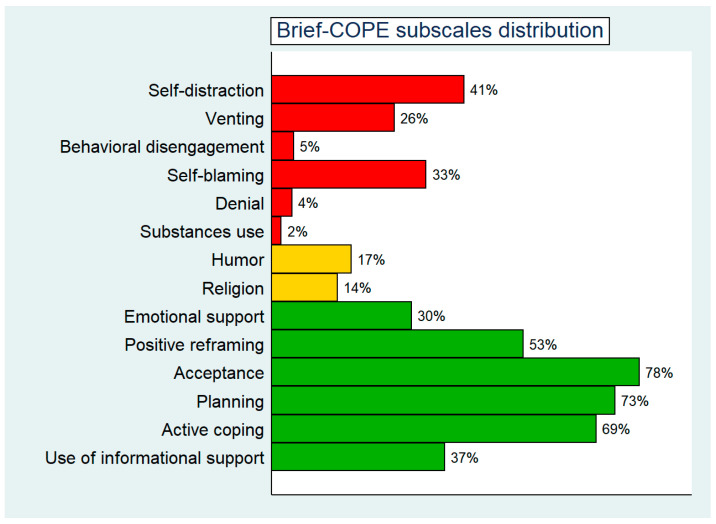
Brief-COPE subscales distribution in the whole sample. Avoidant coping subscales are colored in red, active coping subscales in green, and neither active nor avoidant coping subscales in yellow, respectively. The frequencies for each coping strategy are reported on the right side of each bar as percentages.

An average of 5 Brief-COPE subscales had an above-the-cut-off score (Figure 1).

As explained above, both the problem-focused coping strategy and the emotion-focused strategy measured approach to coping. Within the problem-focused coping strategy: planning, active coping, positive reframing, and the use of informational support were significant; for the strategy of emotion-focused coping: acceptance, self-blaming, emotional support, venting, humor, and religion were significant. Finally, the avoidant coping strategy included self-distraction, behavioral disengagement, denial, and substance use.

### 3.5. Building a Logistic Regression Model of Moderate-to-Severe Anxious Symptoms Predictors and Protectors

In the univariate analysis (Table 2), moderate-to-severe anxiety was found to be positively associated with a lack of religious belief, job changes, changes in work hours, night sleep, and in alcohol consumption, reduction of physical activity, recent RPHSM for a family member, non-vaccinated for COVID-19, psychotropic drug, and psychological treatment. Among coping strategies, moderate-to-severe anxiety was found to be positively associated with self-distraction, venting, use of informational support, denial, humor, behavioral disengagement, emotional support, substance use, and self-blame.

In the univariate analysis, moderate-to-severe anxiety was negatively associated with age ≥ 60 and > 42, male sex, married, living with a partner or partner and children, being occupied, >7-h-night-sleep, being physically active, and fully vaccinated. Among coping strategies, moderate-to-severe anxiety was negatively associated with acceptance.

A positive independent association with moderate-to-severe anxiety (Table 3) was found for a recent RPHSM for a family member, night sleep modifications, psychotropic drug, psychological treatment, and the following coping strategies: self-distraction, venting, behavioral disengagement, emotional support, and self-blame. A negative independent association was found for age > 42, male sex, >7-h-night-sleep, being physically active, and the following coping strategies: positive reframing, and acceptance.

### 3.6. Building a Logistic Regression Model of Depressive Symptoms Predictors and Protectors

In the univariate analysis (Table 4), depressive symptoms were positively associated with a lack of religious belief, changes in job, night sleep, and alcohol consumption, reduction in physical activity, psychotropic drugs, and psychological treatment. Among coping strategies, depression was positively associated with the following coping strategies: self-distraction, venting, use of informational support, denial, humor, behavioral disengagement, emotional support, substance use, and self-blame.

In the univariate analysis, depressive symptoms were negatively associated with age ≥ 60 and age > 42, male sex, being married, parenthood, living with a partner (and children), being occupied, >7-h-night-sleep, being physically active, and being fully vaccinated. Among the coping strategies, depression was negatively associated only with planning.

A positive independent association with depression (Table 5) was found for lack of religious belief, night sleep modifications, psychotropic drugs, psychological treatment, and the following coping strategies: venting, denial, humor, behavioral disengagement, substance use, and self-blame. A negative independent association was found for male sex, parenthood, being occupied, >7-h-night-sleep, being physically active and planning coping strategy.

**Figure 2:**
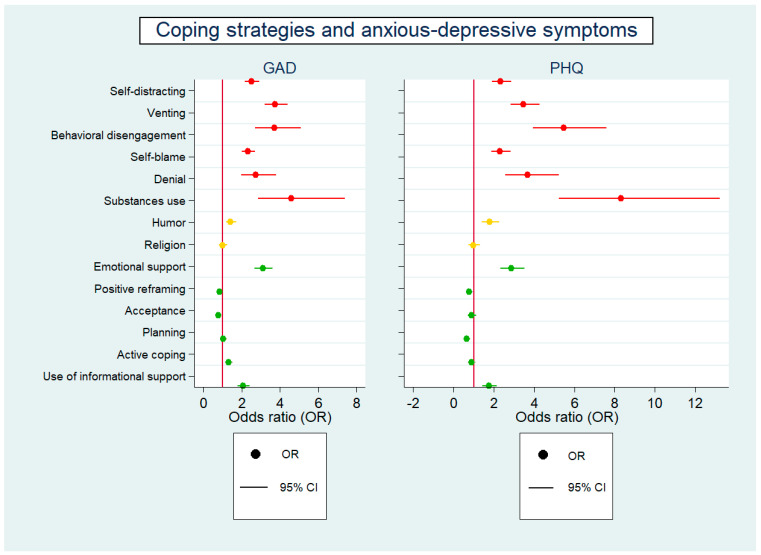
Logistic regression of anxiety and depressive symptoms as predicted by coping styles. Two models are presented for anxiety and depressive symptoms indexed as having reached the GAD−7 and PHQ−9 cut-off values, respectively. Avoidant coping subscales are plotted in red, active coping subscales in green, and neither active nor avoidant in yellow. The odds ratio (OR) indicates the effect size and is reported with 95% confidence intervals (CI). The reference line of odds ratio (OR = 1) is cranberry colored.

As summarized in Figure 2, coping strategies in the avoidance category were significantly associated with higher levels of anxiety and depressive symptoms. The results for the approaching category were more complex. Some strategies, such as emotional support and the use of informational support, were associated with higher levels of both anxiety and depressive symptoms, while others, such as positive reframing, were associated with lower levels of anxiety or depression. Other strategies belonging to the approaching category were found to be significantly associated with lower levels of anxiety (acceptance) rather than depressive symptoms (positive reframing and planning) symptoms. Strategies in the “neither/or” category also displayed mixed associations; humor was significantly associated with higher levels of anxiety and depressive symptoms, while the use of religion did not show any statistically significant association.

## 4. Discussion

The current study aimed to investigate the association between anxiety and depressive symptoms and risk and resilience factors, including coping strategies in the general population during the second wave of the COVID-19 pandemic. Anxiety and depression rates were increased relative to the general prevalence of these disorders. Individual non-modifiable factors (demographics) and modifiable ones (lifestyle, COVID impact, psychiatric treatment) were identified as predictors. Coping strategies contribute to modulating anxiety and depression after the COVID-19 pandemic, with approaching strategies generally mitigating and avoiding strategies aggravating the severity of these psychiatric symptoms.

Within the participants, 27.5% showed significant anxiety symptoms, and 12% met the depression criteria, respectively. The rates of anxious-depressive symptoms found in the current study are lower than in other studies conducted in early 2020, at the beginning of the pandemic [33,34] but consistent with the other investigations conducted during the pandemic [8,17,18,35,36]. A later stage of the pandemic and the success of the vaccination campaign at the time of the study could explain this difference.

Demographic factors play an important role in anxiety and depression. In our study, age above 42 years was found to be protective against anxiety. This result is in line with the previous literature that identified younger adults as the age group with the highest risk of developing psychopathology [17,37,38,39], while older people, although at greater clinical risk, were the least vulnerable. The protective effect of older age could result from their long experience of situations comparable in terms of distress [40]. In addition, men had lower anxiety and depression in keeping with other studies during the COVID-19 pandemic [14,41]. This sex-specific difference could be due not only to the different epidemiology of affective disorders but also to a greater propensity of women to express their discomfort and ask for help [42,43] and their willingness to participate in this type of survey [44].

Lifestyle characteristics were associated with anxiety and depressive symptoms. Consistent with our study, the previous literature has shown that moderate to vigorous physical activity reduces the chances of developing depression and anxiety by 12–32% and 15–34%, respectively [45]. The lack of religious belief predicted depression. Coping strategies involving religion appear to have a strong protective effect after an adverse event [46,47,48]. Opposite findings have also been reported, with higher levels of depression, anxiety, and distress associated with coping strategies involving religion [47,48,49]. The complex effects of religious belief can be explained by considering all aspects of religious dimensions related to coping processes and their distinct effectiveness [50]. Indeed, collaborative religious coping (e.g., the feeling that the person is active and acting with God) appeared to be more closely associated with positive outcomes than others (such as deferring) [47,48,49]. Parenthood has proven to be a protective factor against depressive symptoms. To our knowledge, this is the first study to reveal an apparent protective effect of parenthood on depression during the COVID-19 pandemic. Previous investigations found an increase in anxiety and depression in specific samples of parents [51]. Having a job, as emerged in the current study, was a protective condition against the development of depression. Indeed, work activities can help in emotional regulation thus distracting from epidemic-related information [52]. Furthermore, perceived job insecurity, job changes, loss of employment, and fear of unemployment are significant concerns that contribute to negative affective states, including peritraumatic distress and mood disorders [3,53,54,55]. The previous literature found greater anxiety in people with a recent restraint or with a family member in quarantine, which is consistent with our finding of a negative effect of a family member with recent RPHSMs [1,56,57]. Additionally, a recent change in sleep habits was predictive of increased anxiety and depressive symptoms; conversely, a daily sleep duration greater than 7 h was a protective factor. In line with our results, other studies have found an association between poor sleep quality and peritraumatic distress [3] and affective symptoms [58,59].

Understandably, people who had used psychiatric drugs or sought some form of psychological support showed a higher level of anxiety and depression, which is consistent with previous studies on psychiatric disorders during the pandemic [41,60].

Our study revealed that approach coping strategies were protective against anxiety and depressive symptoms. In particular, positive reframing and acceptance strategies were associated with a nearly 30% reduced risk of anxiety, while the planning strategy had an approximately 40% reduced chance of having depression. Conversely, avoidance coping strategies predicted higher levels of anxiety and depression. More specifically, behavioral disengagement and venting strategies predicted about 2.5 times higher risk of anxiety, while self-distraction, emotional support, and self-blame predicted approximately 1.5-times higher risk of anxiety. Substance use showed the strongest association with higher levels of psychopathology; similarly, venting, denial, and behavioral disengagement showed a 2.5-fold increased risk of depression. Humor and self-blame had an approximately 1.5-fold increased risk of depression. Notably, three avoidant coping strategies including behavioral disengagement, self-blame, and venting were found to predict a higher risk of anxiety and depression.

Differently from the COMET study [10], in which adaptive coping strategies did not have a protective impact on anxiety and depression, our results confirmed and extended the findings of a Greek study, with more benign COVID-19 manifestations [18]. The latter report highlighted that a higher score on the positive coping strategy was associated with a lower prevalence of depression, while more supportive/distractive strategies played the opposite role [18]. The only exception was the emotional support coping, included within the approaching styles, which was associated with greater anxiety in our sample, thus confirming the results of a recent study [61]. A possible explanation for this result is the difficulty of obtaining valid emotional support due to social restrictions during the pandemic, which may have turned the predominant use of emotional support into a dysfunctional strategy.

Finally, humor coping, which is neither an “approach” nor an “avoidant” coping strategy, was significantly associated with a higher risk of depression. Although previous literature reported an association between the latter coping strategy and a reduced level of stress [62], in other reports, conducted during the pandemic, an association between depression and humor emerged [63]. These findings could be explained by using humor and sarcasm as a “relief valve” in subjects experiencing depression [63].

These findings support the theory that approaching coping styles are generally associated with lower practical adjustment, better physical health outcomes, and more stable emotional responses [7,13,64,65,66,67,68]. Conversely, in line with our study (Figure 2), avoidant strategies, including denial, substance, self-blame, and behavioral disengagement, were associated with increased anxiety and depression [61,69,70].

Important strengths of the current study are a large number of participants and the type of recruitment. Most large studies recruited participants using web-based surveys and not directly through the distribution of a questionnaire at a COVID-19 vaccination point of care. This approach allows including populations generally more reluctant to participate in epidemiological surveys during the pandemic, such as the elderly and those who have difficulties with or are skeptical toward web technologies. However, this report suffers from some limitations. First, the cross-sectional design limits the possibility of drawing causal inferences from the results. Second, the study was carried out in a single region, so the findings could be representative only on a regional basis despite the large sample size. Third, the current work did not include the population who decided not to undergo anti-COVID-19 vaccination, which is a minority in Veneto (https://www.regione.veneto.it/dati-vaccinazioni/ (accessed on 21 October 2022)). Future studies are needed including the vaccine-reluctant population.

## 5. Conclusions

Overall, the present study highlighted the impact that coping strategies have, in addition to sociodemographic and lifestyle factors, on the development of anxiety-depressive symptoms associated with the pandemic in the general population. It could be useful for policy-makers to allocate resources to improve supportive, psychotherapeutic, and psychosocial interventions to limit the long-term detrimental effects of the pandemic. Indeed, psychotherapy has been shown to promote adaptive and reduce maladaptive coping strategies for anxiety and depression [69,70,71,72] as well as for psychosocial outcomes [73]. Additionally, preventive measures should be tailored to specific sociodemographic factors such as gender, age, and parental status, and take into account the impact of lifestyle factors on emotional well-being. This includes factors such as changes in sleep patterns, exercise, employment status, religious beliefs, as well as any past or current use of psychotropic medications or psychological counseling.

## Figures and Tables

**Table 1:** Sociodemographic, life habits, GAD-7, and PHQ-9 scores at the time of completion of the questionnaires.

Socio-Demographic Features	n (Number)	% (Percentage)	Life Habit Features	n (Number)	% (Percentage)
Age class (years)			Night sleep (hours/night)		
13–29	902	25.7	<5	185	5.3
30–59	2057	58.6	5–7	2198	62.6
60–99	550	15.7	>7	1100	31.3
			No answer	26	0.7
Sex					
Male	1621	46.2	Change in sleep hours		
Female	1888	53.8	Increased	543	15.5
			Reduced	804	22.9
Marital status			Unmodified	2161	61.6
Married	1450	41.3			
Unmarried	1202	34.2	Physical activity (hours/week)		
Single	580	16.5	None	899	25.6
Separate	98	2.8	<3	1044	29.7
Divorce	119	3.4	3–5	1037	29.5
Widow/er	60	1.7	6–10	403	11.5
			>10	107	3.0
Parental status			No answer	19	0.5
No children	1932	55.1			
One child	642	18.3	Change in physical activity hours		
Two or more children	935	26.6	Increased	712	20.3
			Reduced	1327	37.8
Educational level			Unmodified	1470	41.9
Middle school	378	10.8			
High school	1538	43.8	Alcohol consumption		
Bachelor	432	12.3	Never	937	26.7
Master’s degree	789	22.5	Up to 1 unit daily	2150	61.3
Postgraduate studies	372	10.6	Up to 2 units daily	293	8.3
			More than 2 units daily	70	1.9
Hospitalized relative or friend due to COVID-19?			No answer	59	1.7
Yes	1006	28.8	Change in alcohol consumption		
No	2486	71.2	Increased	342	9.7
			Reduced	569	16.2
Any relative or friend dead from COVID-19 consequences?			Unmodified	2598	74.0
Yes	514	14.7			
No	2979	85.3			
			Religion		
Any psychopharmacological treatment in the last 12 months?			Christian	2537	72.3
No	2831	87.1	Muslim	17	0.5
Yes, on a regular basis	186	5.7	Other religion	46	1.3
Yes, for short periods only	233	7.2	No religious belief	884	25.2
			No answer	25	0.7
Psychotherapy or psychological support in the last 12 months?			Reduced	733	22.4
No	2798	86.1	Change in work habits:		
No, but some form of psychological support was present	105	3.2	No	1880	53.6
Yes	347	10.7	Switch to smart working	1444	41.1
			Job loss during the pandemic	185	5.3
			Occupational status		
			Unemployed	230	6.5
			Employed	2309	65.8
			Retired	301	8.6
			Student	669	19.1
GAD-7, mean score 6.8 (SD = 5.3)			PHQ-9, average score 6.1 (SD = 5.5)		
Minimal anxiety (0–4)	1425	40.6	Negative	3074	87.6
Mild anxiety	1119	31.9	Positive	435	12.4
Moderate anxiety	586	16.7			
Severe anxiety	379	10.8			

**Table 2:** Univariate logistic regression analysis of factors associated with moderate-to-severe anxiety (GAD-7 score ≥ 10).

Predictors	OR (95% CI)	*p*
Age > 42 years	0.38 (0.32–0.44)	<0.001 ***
Age ≥ 60 years	0.39 (0.30–0.50)	<0.001 ***
Sex (male)	0.51 (0.44–0.60)	<0.001 ***
Marital status (married)	0.50 (0.43–0.59)	<0.001 ***
Family status (living with partner or partner and children)	0.53 (0.46–0.62)	<0.001 ***
Parenthood	0.50 (0.43–0.58)	<0.001 ***
Educational level (bachelor’s degree or higher)	1.01 (0.94–1.26)	0.257
Lack of religious belief	1.63 (1.38–1.92)	<0.001 ***
Job status (occupied)	0.68 (0.58–0.79)	<0.001 ***
Job changes (job loss or switch to remote working)	1.71 (1.47–1.98)	<0.001 ***
Health worker	0.68 (0.46–1.01)	0.057
Night sleep (>7 h/night)	0.68 (0.57–0.80)	<0.001 ***
Physical activity (any level of activity)	0.65 (0.55–0.77)	<0.001 ***
Alcohol consumption	0.86 (0.73–1.01)	0.074
Work hours modified (increased or decreased)	1.59 (1.37–1.84)	<0.001 ***
Modified night sleep (increased or decreased)	3.18 (2.73–3.71)	<0.001 ***
Decreased physical activity	1.42 (1.22–1.66)	<0.001 ***
Alcohol consumption modified (increased or decreased)	1.82 (1.54–2.14)	<0.001 ***
At least one relative hospitalized due to COVID-19	1.21 (1.03–1.42)	0.020 *
At least one relative lost due to COVID-19	1.14 (0.93–1.40)	0.210
Restrictive Public Health and Social Measures (in the last 10 days)	2.12 (1.43–3.12)	<0.001 ***
No previous doses of anti-COVID vaccine	1.35 (1.12–1.64)	0.002 **
Already fully vaccinated	0.61 (0.52–0.72)	<0.001 ***
Psychotropic drug use	3.62 (0.45–0.86)	<0.001 ***
Psychological support	3.99 (3.25–4.89)	<0.001 ***
Coping subscales		
Positive reframing	0.85 (0.73–0.99)	0.033 *
Self-distraction	2.51 (2.16–2.92)	<0.001 ***
Venting	3.75 (3.19–4.40)	<0.001 ***
Use of informational support	2.08 (1.79–2.42)	<0.001 ***
Active coping	1.31 (1.11–1.54)	0.001 **
Denial	2.74 (1.98–3.79)	<0.001 ***
Religion	1.01 (0.81–1.25)	0.940
Humor	1.42 (1.18–1.72)	<0.001 ***
Behavioral disengagement	3.70 (2.65–3.63)	<0.001 ***
Emotional support	3.10 (2.65–3.63)	<0.001 ***
Substance use	4.60 (2.86–7.40)	<0.001 ***
Acceptance	0.78 (0.66–0.93)	0.007 **
Planning	1.03 (0.87–1.22)	0.689
Self-blame	2.32 (1.99–2.71)	<0.001 ***

Statistical significance (* *p* < 0.05, ** *p* < 0.01, and *** *p* < 0.001).

**Table 3:** Multivariate logistic regression analysis of factors associated with moderate-to-severe anxiety (GAD-7 Score ≥ 10).

Predictors	OR (95% CI)	*p*
Age > 42 years	0.50 (0.41–0.61)	<0.001 ***
Sex (men)	0.75 (0.62–0.90)	0.002 **
Restrictive Public Health and Social Measures in the last 10 days	2.01 (1.31–3.34)	0.002 **
Night sleep (>7 h/night)	0.67 (0.54–0.82)	<0.001 ***
Physical activity (at any level of activity)	0.66 (0.54–0.81)	<0.001 ***
Modified night sleep (increased or decreased)	2.26 (1.88–2.71)	<0.001 ***
Psychotropic drug use	2.73 (2.10–3.54)	<0.001 ***
Psychological support	1.76 (1.37–2.26)	<0.001 ***
Coping subscales		
Positive reframing	0.72 (0.60–0.87)	0.001 **
Self-distraction	1.67 (1.38–2.01)	<0.001 ***
Venting	2.43 (1.98–2.98)	<0.001 ***
Behavioral disengagement	2.54 (1.60–3.80)	<0.001 ***
Emotional support	1.55 (1.26–1.90)	<0.001 ***
Acceptance	0.73 (0.59–0.92)	0.006 **
Self-blame	1.43 (1.18–1.74)	<0.001 ***

Statistical significance (** *p* < 0.01, and *** *p* < 0.001).

**Table 4:** Univariate logistic regression analysis of factors associated with depressive symptoms at PHQ-9 (total score > 10, at least 5 items ≥ 2, and item 1 or 2 ≥ 2).

Predictor	OR (95% CI)	*p*
Age > 42 years	0.36 (0.29–0.45)	<0.001 ***
Age ≥ 60 years	0.54 (0.39–0.75)	<0.001 ***
Sex (men)	0.52 (0.42–0.65)	<0.001 ***
Marital status (married)	0.43 (0.34–0.54)	<0.001 ***
Family status (living with partner or partner and children)	0.39 (0.32–0.48)	<0.001 ***
Parental status	0.38 (0.30–0.48)	<0.001 ***
Educational level (bachelor’s degree or higher)	0.77 (0.63–0.94)	0.012 *
No religious belief	2.30 (1.86–2.83)	<0.001 ***
Job status (occupied)	0.40 (0.32–0.49)	<0.001 ***
Job changes (job loss or switch to remote-working)	1.66 (1.36–2.03)	<0.001 ***
Health worker	0.53 (0.28–0.99)	0.046 *
Night sleep (>7 h/night)	0.63 (0.50–0.81)	<0.001 ***
Physical activity (any level of activity)	0.58 (0.47–0.71)	<0.001 ***
Alcohol consumption	0.82 (0.66–1.03)	0.087
Work hours modified (increased or decreased)	1.24 (1.01–1.51)	0.038 *
Modified night sleep (increased or decreased)	3.86 (3.12–4.78)	<0.001 ***
Decreased physical activity	1.53 (1.25–1.88)	<0.001 ***
Alcohol consumption modified (increased or decreased)	1.70 (1.37–2.10)	<0.001 ***
At least one relative hospitalized due to COVID-19	1.01 (0.81–1.27)	0.898
At least one relative lost to COVID-19	1.07 (0.81–1.41)	0.634
Restrictive Public Health and Social Measures (in the last 10 days)	1.88 (1.16–3.03)	0.010 *
No previous doses of anti-COVID vaccine	1.16 (0.90–1.51)	0.246
Already fully vaccinated	0.65 (0.52–0.82)	<0.001 ***
Coping subscales		
Psychotropic drugs use	4.41 (3.46–5.62)	<0.001 ***
Psychological support	4.36 (3.44–5.54)	<0.001 ***
Positive reframing	0.77 (0.63–0.94)	0.011 *
Self-distraction	2.35 (1.91–2.88)	<0.001 ***
Venting	3.48 (2.83–4.28)	<0.001 ***
Use of informational support	1.75 (1.43–2.14)	<0.001 ***
Active coping	0.89 (0.72–1.11)	0.306
Denial	3.67 (2.58–5.23)	<0.001 ***
Religion	0.98 (0.73–1.31)	0.884
Humor	1.78 (1.40–2.26)	<0.001 ***
Behavioral disengagement	5.47 (3.94–7.60)	<0.001 ***
Emotional support	2.88 (2.34–3.53)	<0.001 ***
Substance use	8.31 (5.22–13.23)	<0.001 ***
Acceptance	0.90 (0.71–1.14)	0.374
Planning	0.66 (0.54–0.82)	<0.001 ***
Self-blame	2.32 (1.89–2.84)	<0.001 ***

Statistical significance (* *p* < 0.05, and *** *p* < 0.001).

**Table 5:** Multivariate logistic regression analysis of factors associated with depression at PHQ-9 (Total score > 10, at least 5 items ≥ 2, and item 1 or 2 ≥ 2).

Predictor	OR (95% CI)	*p*
Sex (male)	0.65 (0.50–0.85)	0.002 **
Parental status	0.65 (0.49–0.86)	0.003 **
No religious belief	1.55 (1.18–2.04)	0.002 **
Job status (occupied)	0.44 (0.34–0.57)	<0.001 ***
Night sleep (>7 h/night)	0.62 (0.47–0.83)	0.001 **
Physical activity (at any level of activity)	0.55 (0.42–0.72)	<0.001 ***
Modified night sleep (increased or decreased)	2.35 (1.83–3.03)	<0.001 ***
Psychotropic drug use	2.98 (2.20–4.04)	<0.001 ***
Psychological support	2.02 (1.50–2.74)	<0.001 ***
Coping subscales		
Venting	2.43 (1.88–3.15)	<0.001 ***
Denial	2.57 (1.61–4.11)	<0.001 ***
Humor	1.58 (1.16–2.14)	0.003 **
Behavioral disengagement	2.31 (1.48–3.64)	<0.001 ***
Substance use	3.33 (1.74–3.38)	<0.001 ***
Planning	0.58 (0.44–0.77)	<0.001 ***
Self-blame	1.57 (1.21–2.05)	0.001 **

Statistical significance (** *p* < 0.01, and *** *p* < 0.001).

## Data Availability

The data that support the findings of this study are available on request from the corresponding author. The data are not publicly available due to ethical reasons.

## Supplementary Materials

The following supporting information can be downloaded at: https://www.mdpi.com/article/10.3390/ijerph20042974/s1.
