# Supplementary material for: Anxiety and Depression during the Second Wave of the COVID-19 Pandemic: The Role of Coping Strategies
Source: Int J Environ Res Public Health. 2023 Feb 8;20(4):2974. doi: 10.3390/ijerph20042974 (PMC9957361; doi:10.3390/ijerph20042974)
Supplement: Supplementary file 1 [file ijerph-20-02974-s001.zip › ijerph-2182448-supplementary.pdf]

Supplementary material

Anxiety and Depression during the Second Wave of the COVID-19 Pandemic: The Role of Coping Strategies

Alessandro Miola[1,2,3*], Stefano Caiolo[*2,4], Giancarlo Pontoni[5*], Erica Pozzan[2], Chiara Moriglia[6], Filippo Simionato[2], Sergio Garofalo[4], Giulia Perini[1,2,3], Fabio Sambataro[1,2]

1- Department of Neuroscience (DNS), Padua Neuroscience Center, University of Padova, Padua, Italy
2- University of Padova, Padua, Italy
3- Casa di Cura Parco dei Tigli, Padua, Italy
4- Psychiatry Section - Military Department of Forensic Medicine, Padua, Italy
5- Psychiatry Section, Psychophysiological Selection Office, Italian Army National Recruitment and Selection Center, Foligno (PG), Italy
6- Psyops Development Center, 28th (APICE) Regiment, Pavia, Pesaro, Italy

* These authors contributed equally to this work

Corresponding author:
Fabio Sambataro
Department of Neuroscience (DNS), University of Padova
Via Belzoni 160, I-35121 Padova
Tel: +390498211980
Email: Fabio.sambataro@unipd.it

Sample size calculation

The sample size estimation was performed using Raosoft software (http://www.raosoft.com/samplesize.html). The minimum number of subjects required for this study was 644 considering the 5% margin of error, a confidence level of 99%, a response distribution of 50%, and the reference population width of 4879133 inhabitants (data from Veneto population in January 2020 by the Italian National Institute of Statistics, ISTAT). Given 75% as the percentage of valid answers, our estimate was 830 people to be recruited for this study at least.

Anti-COVID-19 vaccine and clinical features

At the time of the questionnaire, the first of two anti-COVID-19 vaccine doses was administered to 17% (n=593) of the participants, while in 2.5% of cases (n=92) the vaccine cycle was completed with only one dose (due to a previous SARS-CoV-2 infection or a single-dose type of vaccination). In 47% of the cases (n=1645) participants had already received the second dose and in 33.5% (n=1179) the anti-COVID-19 booster. Twenty-five percent of the participants (n=876) were concerned about the administration of the vaccine. In 14% of cases (n=499), participants would have preferred not to be vaccinated. When asked about their satisfaction with the anti-COVID-19 vaccine information campaign, participants reported being unsatisfied in 40% of cases (n=1415). Only 7% (n=230) of the sample admitted not being informed about the vaccine. The most frequent source of information used was television in 55% (n=1932) of the cases, followed by scientific articles in 47% (n=1647), general practitioners in 30% (n = 1048) and social networks in 28% (n=972) of the cases.

Typical symptoms of COVID-19 were reported in 80% (n=218) of the tested positive participants. Symptoms were described as light (only fever, ageusia/anosmia, headache) in 78% (n=178), intermediate (respiratory or cardiac involvement) in 17% (n=38), severe (hospitalization required) in 5% (n=12) of the cases. Most of the tested positive patients (61%, n=162) were treated at home, otherwise, 3% (n=8) were hospitalized in an ordinary ward and 2% of cases (n=5) needed intensive care hospitalization.
